# How Egg Storage Duration Prior to Incubation Impairs Egg Quality and Chicken Embryonic Development: Contribution of Imaging Technologies

**DOI:** 10.3389/fphys.2022.902154

**Published:** 2022-05-31

**Authors:** Hans Adriaensen, Vanille Parasote, Ines Castilla, Nelly Bernardet, Maeva Halgrain, François Lecompte, Sophie Réhault-Godbert

**Affiliations:** ^1^ INRAE, CNRS, IFCE, Université de Tours, PRC, Nouzilly, France; ^2^ INRAE, CHU de Tours, Université de Tours, PIXANIM, Nouzilly, France; ^3^ INRAE, Université de Tours, BOA, Nouzilly, France

**Keywords:** chicken, egg, storage, duration, egg quality, embryonic development, magnetic resonance imaging, computed tomography

## Abstract

Storing fertilised eggs prior to incubation is a frequent practice in commercial hatcheries to coordinate activities and synchronise hatchings. However, the conditions used to store eggs can have major impacts on egg quality and the subsequent viability of chicken embryos. While storage temperatures of 16–18°C are classically used in hatcheries, the duration of storage varies from three to more than 10 days. We explored the effect of storage duration (zero, three or 10 days; D0, D3 and D10, respectively) at 16°C, 80% relative humidity (RH) on egg quality (Broiler, Ross 308), using computed tomography (CT) and classical measurements (egg weight, eggshell strength, egg white pH, Haugh units, yolk index and colour). The results revealed that a storage duration of up to 10 days negatively affected some egg quality traits (yolk index and volume, air chamber volume and egg white pH). Eggs stored for three or 10 days were further incubated for 11, 13 or 15 days (37.8°C, 55% RH). Eggs were analysed by magnetic resonance imaging (MRI) and CT to assess the development of the embryo and internal egg changes occurring during incubation. First, data showed that the fertility and sex ratio of eggs were not affected by storage duration. However, the mortality of viable eggs was increased in the D10 group compared to the D3 group. Results of non-invasive imaging technologies revealed that the storage of eggs for 10 days impaired embryo growth as early as 11 days of incubation (decrease in brain and embryo volumes). Collectively, these data provide new evidence that the duration of egg storage negatively affects embryonic growth. They further corroborate that this parameter is likely to be crucial to synchronising embryonic stages and maybe reducing the hatching window, hence limiting the time spent by newborn chicks in hatchers. In addition, our results highlight that CT and MRI imaging technologies are useful non-invasive tools to evaluate egg quality prior to incubation and the impact of storage (or incubation) practices on developmental growth of the embryo.

## Introduction

Egg storage prior to incubation is an common practice in the broiler industry (Fasenko, 2007). It allows coordinating hatchery activities, considering the time between laying and the arrival of eggs in hatcheries. It supplies a certain flexibility towards demands and facilitates synchronisation of hatchings.

**FIGURE 1:**
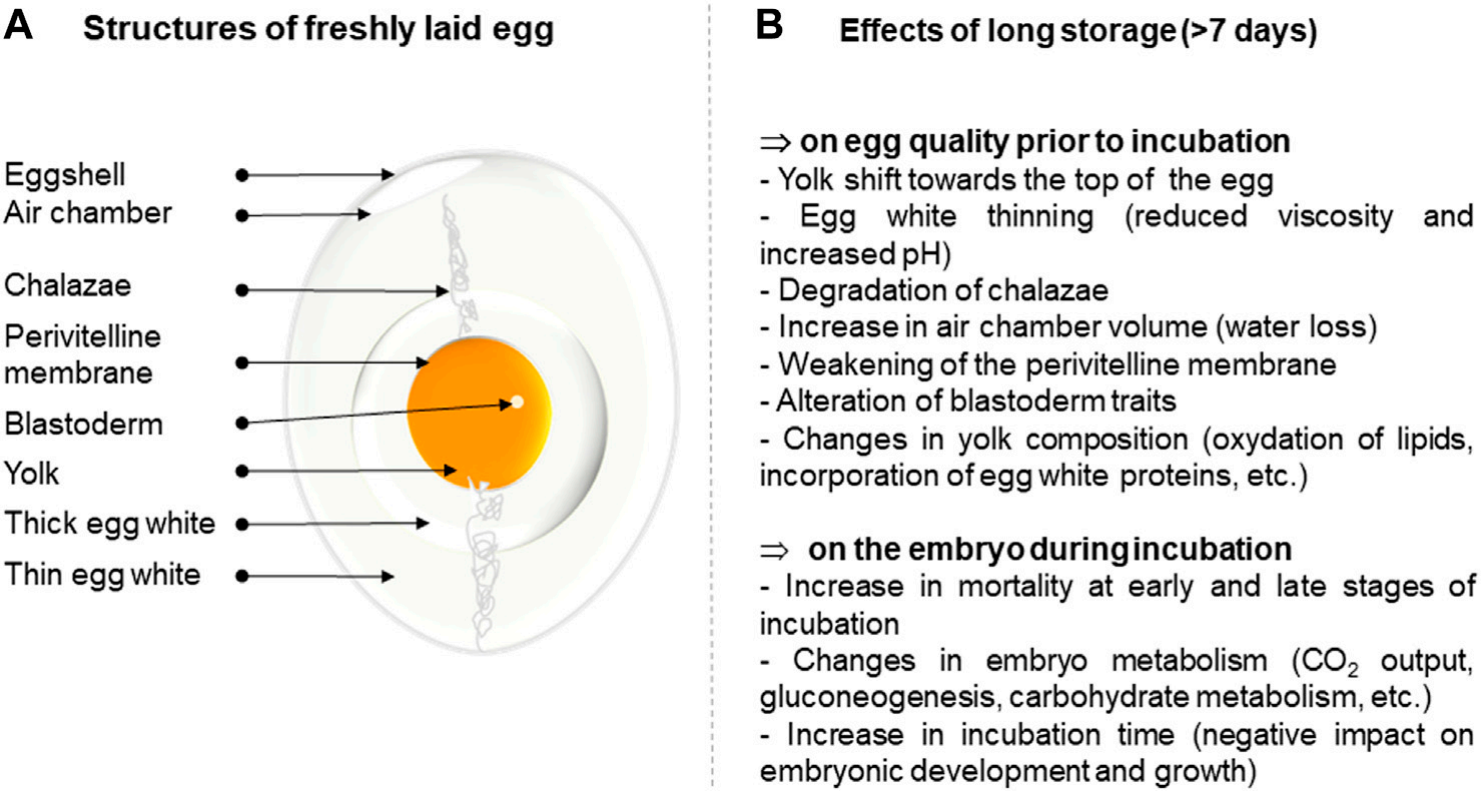
Summary of the effect of long-term storage on egg quality and subsequent development of the embryo. **(A)**. Structure of a freshly laid egg. **(B)**. Effects of long-term storage on egg parameters.

Egg storage prior to incubation does not negatively affect hatchability when the duration of storage does not exceed 7 days (Fasenko, 2007). It is noteworthy that incubating freshly laid eggs is, unexpectedly, not associated with higher embryo viability compared to that of stored eggs, and eggs incubated the day of laying tend to hatch later compared with eggs stored for one or 2 days (Reis et al., 1997). The freshly laid egg contains a high concentration of carbon dioxide that may be detrimental to initiating the first stages of embryo development, while the thickness of the egg white is assumed to slow vital gas diffusion and limit access to egg nutrients (Benton and Brake, 1996). On the other hand, extended storage can have a dramatic impact on blastoderm reactivation, even if it is conducted at 17–18°C under controlled relative humidity. Storage beyond 7 days is usually associated with decreased hatchability rates compared to short periods of storage (Lapão et al., 1999; Elibol et al., 2002; Hamidu et al., 2011; Goliomytis et al., 2015; Bakst et al., 2016b; Abioja et al., 2021) and egg storage time was evidenced to be the most important factor (among genotype, hen age, setter and hatcher type) associated with early embryonic mortality (Grochowska et al., 2019). Long storage induces an alteration of many egg quality features including a decrease in yolk and albumen quality parameters and water loss, but also impairs the quality of the blastoderm (increased diameter, small shift of its position on the yolk likely due to the progressive disintegration of the chalazae, decreased number of viable cells, increased necrosis and apoptosis, etc.) (Burkhardt et al., 2011; Bakst et al., 2012; Abioja et al., 2021) (Figure 1). These combined alterations result in an increase of early and late embryo mortality. Similar observations have been reported in the literature for other domestic avian species (Fasenko et al., 2001; Hassan et al., 2005; Nowaczewski et al., 2010; Hyánková and Novotná, 2013; Kouame et al., 2019; Taha et al., 2019).

Upon egg storage at cooled temperature, the embryo metabolism changes (Christensen et al., 2001) and the embryonic development pauses. The embryo (blastoderm) enters a temperature-induced diapause (Pokhrel et al., 2021), also termed dormancy, which is characterised by reduced cellular activity and suppressed apoptosis (Tona et al., 2003b; Bakst et al., 2016b; Ko et al., 2017). However, lengthening the storage period of eggs irreversibly impairs embryo survival. Prolonged storage (over 10 days) has been shown to activate mechanisms of apoptotic cell death at the blastodermal level (upregulation of pro-apoptotic genes), resulting in decreasing blastodermal cell viability (Hamidu et al., 2011). Long storage also affects the ability of the embryo to resume development once incubated, while embryos that survive long storage treatment undergo delayed hatching by several hours (Christensen et al., 2002; Tona et al., 2003a; Tona et al., 2003b). Long storage of eggs can affect the intestinal morphology of the chicks, the expression of nutrient transporters (Yalcin et al., 2016; Yalcin et al., 2017), chick immunocompetence (Goliomytis et al., 2015) and hormonal metabolism (Tona et al., 2003b). Long storage may also have long-term negative effects on the quality and physiology of hatched chicks (Tona et al., 2003a; Tona et al., 2003b; Reijrink et al., 2010; Yalcin et al., 2017; Mróz et al., 2019).

Many studies have been published on the effects of prolonged storage on egg quality and blastoderm characteristics, but also on the chick after hatching. However, only few articles address the impact of egg storage on embryonic development. Long storage conditions have been shown to affect the development of the embryo, which exhibits lower overall weight (Christensen et al., 2002; Hamidu et al., 2011; Bakst et al., 2016a), lighter heart, liver and thigh muscle (Christensen et al., 2002) and smaller leg bones (Yalcin and Siegel, 2003), compared to those stored for one to 4 days. All these observations strongly support that the development of embryos after long storage is slowed down, which likely explains the delayed hatching observed in several studies (Christensen et al., 2002; Tona et al., 2003a; Tona et al., 2003b) when compared to eggs stored for only few days. It seems that embryos from eggs stored for a long period require more time in the incubator to reach the developmental maturity that is necessary for hatching, compared with eggs stored for a short period.

Based on these data, which were mostly obtained after egg opening, we evaluated whether computed tomography (CT) and magnetic resonance imaging (MRI), as non-invasive technologies, could be used to monitor internal changes that occur in eggs upon short and long storage. CT has been previously applied to localise the germinal disc *in ovo* (Bartels et al., 2008) and MRI techniques have been used to study the egg yolk structure (Hutchison et al., 1992), the localisation of the germinal disc (Klein et al., 2002) and to monitor the development of the embryo under conventional conditions (Bain et al., 2007; Boss et al., 2008; Zhou et al., 2015; Lindner et al., 2017; Kantarcioglu et al., 2018). However, the use of such techniques to assess the effect of storage time on egg quality and embryo development has not been reported to date.

## Materials and Methods

**FIGURE 2:**
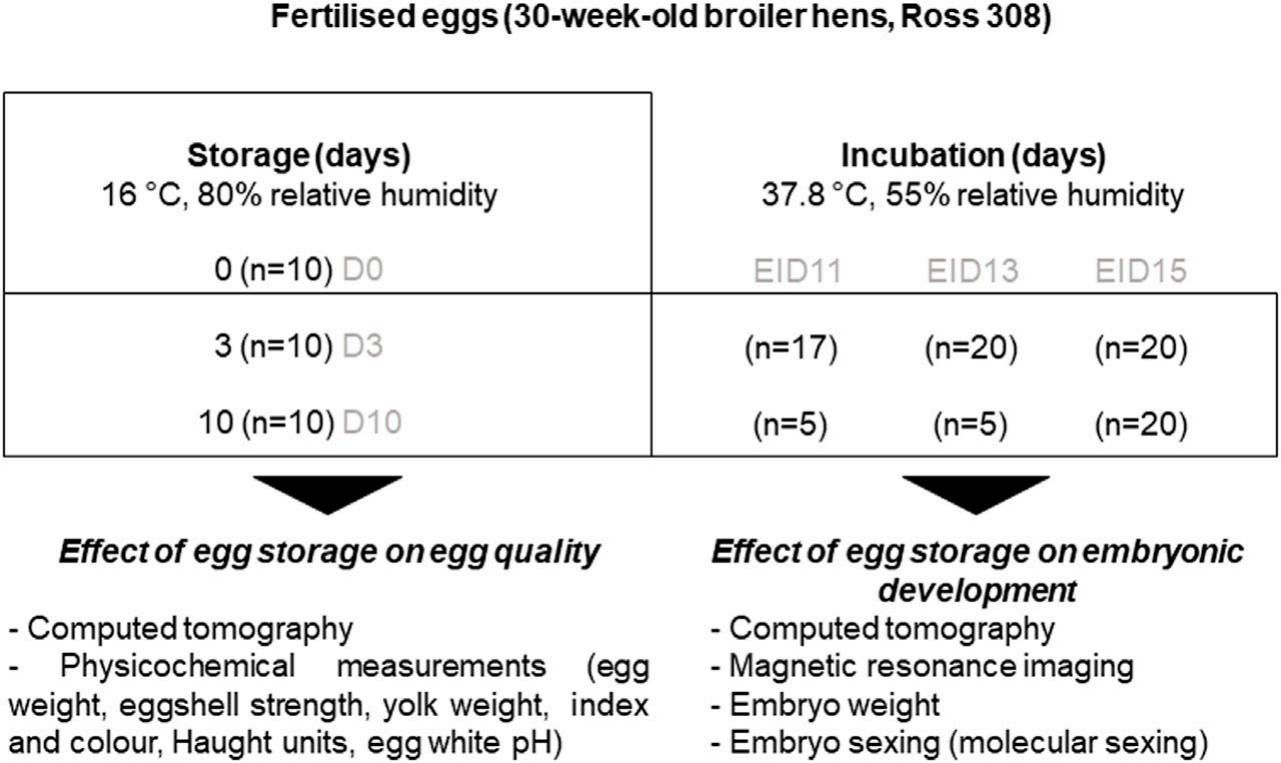
Experimental design. The internal quality of freshly laid eggs (day of laying, D0) and eggs stored for three (D3) or 10 days (D10) was measured to evaluate changes occurring during storage (left panel). A series of eggs stored for three or 10 days were further incubated for 11 (EID11), 13 (EID13) or 15 (EID15) days to analyse the impact of storage on embryonic development (right panel).

The experimental workflow is illustrated in Figure 2. Using CT, we analysed the quality of eggs collected on the day of laying (D0) or after three and 10 days of storage (D3 and D10, respectively) at 16°C, 80% RH. Eggs were then opened and classical egg quality parameters were measured. Remaining D3 and D10 eggs were incubated (37.8°C, 55% RH) for 11, 13 and 15 days and analysed by MRI and CT, followed by egg opening to weigh the embryo.

### Incubation Procedures and Sampling

Freshly laid fertilised eggs were obtained from 30-week-old broiler breeder hens (ROSS 308, Boyé Accouvage, La Boissière en Gâtine, France). Eggs were all weighted and ten-egg batches of similar egg weight (56.5 ± 0.52 g) were formed. The egg weight in each batch ranged from 53 to 60.4 g to illustrate natural egg weight heterogeneity. Ten eggs were kept (D0) for analyses, while the remaining eggs were stored in the Poultry Experimental Facility (PEAT) UE1295 (INRAE, F-37380 Nouzilly, France, DOI: 10.15454/1.5572326250887292E12) in a dedicated room at 16°C, 80% RH for three (D3) or 10 days (D10). Ten eggs were collected at D0, D3 and D10 and were analysed by computed tomography, followed by the measurement of some egg quality parameters. The day before incubation 60 D3 and 60 D10 eggs were placed at room temperature (45% hygrometry), and then transferred into a 3900-egg incubator (Bekoto B64-S, Pont-Saint-Martin, France) set at 37.8°C, 55% RH (automatic turning every hour, large end of eggs on top). After 11, 13 and 15 days of incubation (Embryonic incubation day 11, 13 and 15 or EID13, EID13, and EID15, respectively), D3 and D10 eggs containing viable embryos were analysed by computed tomography while others were selected for MRI analyses. After CT and MRI acquisitions, all eggs were weighed and opened to collect embryos that were killed by decapitation. This experimental procedure meets the guidelines approved by the Institutional Animal Care and Use Committee (IACUC). Dead embryos were weighed and a small piece of the liver was collected, kept at −20°C for molecular sexing.

### Measurements of Egg Quality Parameters During Egg Storage and Incubation (CT and Physicochemical Measurements)

A series of ten eggs were collected the day of laying, three and 10 days after storage. Eggs were analysed by clinical computed tomography (Siemens Somatom^®^ Definition AS 128, Siemens, Germany). The X-ray source was set at 140 kV and 400 mA/s. The image acquisition mode was 16 cm * 16 cm, 512 pixels matrix size with a slice thickness of 0.4 mm and a resolution of 312.5 µm. Safire U 40 and V80 reconstruction filters were used to characterise the internal components of the egg and the shell, respectively (Table 1). The analysis of egg structures was performed with the Syngo. Via software (Siemens, Germany). The volume of the air chamber and the albumen was estimated automatically using ITK Snap software (Yushkevich et al., 2006).

**TABLE 1:** Egg analysis *via* imaging methods.

Traits	Volume [min; max] mm^3^		Imaging method
	During storage (D0, D3, D10)	During incubation (EID11, EID13, EID15)	
Egg white	[31,950; 39,970]	[1,512; 4,664]	CT/T_1_ MRI
Egg yolk	[8,371; 11,740]	N/A	CT/T_1_ MRI
Air chamber	[144.0; 1,110.0]	[3,249; 5,342]	CT
“Grey zone” including the blastoderm^a^	[153.3; 502.9]	N/A	CT
Allantoic fluid	N/A	[4,026; 12,680]	T_2_ MRI
Amniotic fluid	N/A	[3,125; 3,762]	T_2_ MRI
Eyes	N/A	[336.6; 632.6]	T_2_ MRI
Brain	N/A	[260.5; 781.5]	T_2_ MRI
Embryo	N/A	[4,429; 6,524]	T_2_ MRI
Embryo + Yolk	N/A	[14,010; 27,690]	T_2_ MRI

a This trait was estimated based on pixels that could not be assigned to the egg yolk or egg white. N/A. not applicable.

Following CT acquisition, eggs were characterised for their quality. Egg weight, eggshell strength, Haugh units, yolk colour and index were measured using a Digital Egg Tester 6,000 (Nabel, Kyoto, Japan). The egg yolk was weighed and egg white pH was measured (Eutech pH metre, Thermo Fisher Scientific, Illkirch, France).

### MRI Analyses During Incubation

The eggs stored for 3 days (D3) or 10 days (D10) were incubated. After 11, 13 or 15 days (EID11, EID13, EID15), eggs were collected and refrigerated at 4°C for 1 h and 10 min at −20°C prior to analyses with 3 T (T) MRI scanner (Siemens Magnetom^®^, Verio, Erlangen, Germany). Such an egg cooling was necessary to anesthetise the chick and thus avoid movements of the embryos during MRI acquisition.

We used one radio frequency (RF) ‘loop’ coil, with an inner diameter of 7 cm, to analyse eggs independently. Each egg was inserted in the middle of the loop coil.

Two separate MRI image sequences (T_1_: spin-lattice or longitudinal relaxation time and T_2_: spin-spin or transverse relaxation time) were performed on the whole brain, in order to get two distinct image contrasts. The T_1_ 3D and T_2_ 3D were the Magnetisation Prepared Rapid Acquisition Gradient Echo (MPRAGE) and the Sampling Perfection with Application-optimised Contrasts (SPACE), respectively.

The acquisition parameters for these T_1_ and T_2_ anatomical analyses were as follows:

-T_1_ 3D: repetition time (TR) = 1970 ms; echo time (TE) = 3.34 ms; inversion time (TI) = 900 ms; flip angle = 9°; field-of-view (FOV): 81 * 81 mm^2^; matrix: 192 * 192^2^; and a slice thickness of 0.4 mm resulting in a voxel size of 0.42 * 0.42 * 0.40 mm^3^. A bandwidth of 150 Hz/Px and two number of excitations (NEX) producing an acquisition time of 9 min 29 s were used.

-T_2_ 3D: TR = 1860 ms; TE = 140 ms; flip angle = 140°; bandwidth of 296 Hz/Px; and a turbo factor of 99. The inter echo space was 7.38 ms. The FOV was 70 * 70 mm^2^. The matrix was 192 * 192^2^ and slice thickness was 0.35 mm, which ended with a voxel size of 0.36 * 0.36 * 0.35 mm^3^. The acquisition time was 8 min 57 s.

Volumes on the MRI images were estimated based on T_1_ images of the yolk and the albumen, and on the T_2_ images for the brain, eyes, yolk sac, allantoic fluid and embryo, as T_2_ contrast clearly allows distinction of water content between egg compartments (Table 1). At EID13 and EID15, the yolk sac and embryo signals were merged, as the tissue aspect of the yolk sac as a very dense and vascularised tissue (Wong and Uni, 2021) resembled the embryo and both structures could not be distinguished.

For volume estimation, the Digital Imaging and Communication in Medicine (DICOM) images were converted into the Neuroimaging Informatics Technology Initiative (NIfTI) format.

The NIfTI images were read with ITK Snap, which is a free, post-processing software generally used to segment 3D medical image structures (Yushkevich et al., 2006). The segmentation of “area growing” type was done automatically and then corrected manually.

After MRI analyses, eggs were weighed, embryos were removed from the eggs and decapitated and embryo weight was determined. Small pieces of the liver were collected and stored at −20°C for further analysis (molecular sexing).

### Molecular Sexing

Molecular sexing was performed as previously published with minor adjustments (He et al., 2019). Small pieces of EID11, EID13 and EID15 embryo livers were lysed in 150 μl of lysis buffer containing 10% of chelating beads (Chelex 100), 0.2% SDS, 10 mM Tris pH 8 and 0.2 mg/ml Proteinase K). Samples were incubated for 3 hours at 55°C followed by a 15-min incubation at 95°C. Samples were then centrifuged for 3 min at room temperature at maximum speed with a Mini centrifuge 6K (ExtraGene, Taichung City, Taiwan). Supernatants were recovered and stored at −20°C until use. DNA lysate quantification was assessed by reading the 260 nm absorbance with a micro volume spectrophotometer (Nanodrop One Thermo Scientific, Wilmington United States). Embryo lysates were diluted ten times in nuclease free water and 1 μl of dilution was mixed on ice with primer SWIM (forward: 5′- GAG​ATC​ACG​AAC​TCA​ACC​AG -3′/reverse: 5′- CCA​GAC​CTA​ATA​CGG​TTT​TAC​AG -3′), which is female specific and primer 12S (forward-5′ CTA​TAA​TCG​ATA​ATC​CAC​GAT​TCA- 3′, reverse: 5′- CTT​GAC​CTG​TCT​TAT​TAG​CGA​GG -3′) and Dream Taq PCR Master Mix (2X), according to the manufacturer’s recommendations (Thermo Fisher Scientific, Illkirch, France). Amplification by polymerase chain reaction (PCR) was performed using a thermocycler (Eppendorf, Montesson, France), as described previously (He et al., 2019). PCR products were loaded on a 2% agarose gel containing 0.01% gel Red in 1X TAE buffer, and separated by electrophoresis at 100 V. Gels were imaged using a Bio-Print imager (Vilber Lourmat, Marne-la-Vallée, France). Female samples exhibited two amplification products (131 bp for 12S and 212 bp for SWIM gene), while male samples exhibited only one amplification product (131 bp for 12S gene).

### Statistical Analyses

All statistical analyses were performed using XLSTAT software (Data Analysis and Statistical Solution for Microsoft Excel, Addinsoft, Paris, France 2017). For most parameters, normality of the samples was not achieved (Shapiro–Wilk test). Thus, all statistical analyses (except for embryo weight) were performed using a Kruskal–Wallis test (*p* < 0.05), followed by a pair comparison using a Mann–Whitney test (*p* < 0.05), when required. For embryo weight, we used an ANOVA test.

## Results

### Impact of Egg Storage for Zero, Three or Ten Days on Egg Quality

**FIGURE 3:**
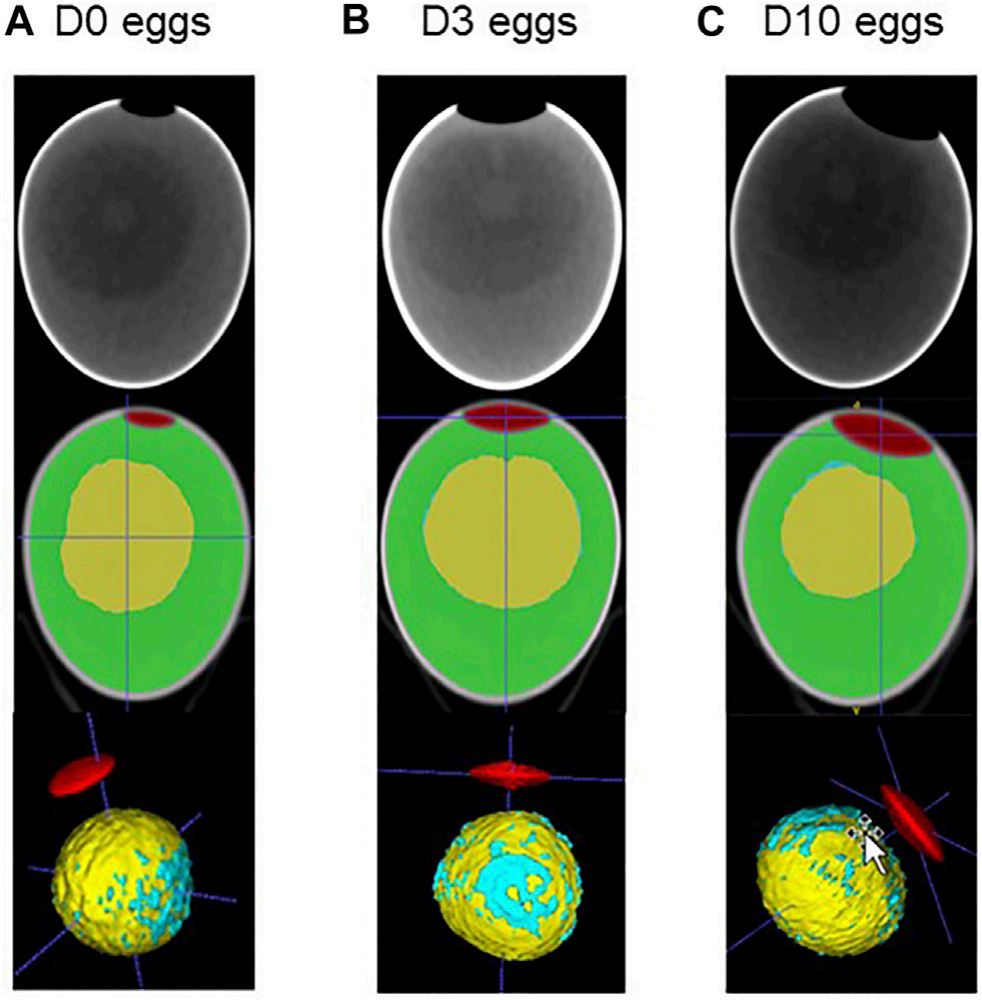
CT imaging of fertilised eggs stored for zero, three or 10 days (D0 **(A)**, D3 **(B)** and D10 **(C)**, respectively). Using ITK. snap tool (Yushkevich et al., 2006), images obtained after CT imaging (upper panel) were analysed for 3D reconstruction, segmentation and coloured for illustration. The air chamber is illustrated in red, the yolk is shown in yellow, the white in green and the blastoderm in blue. It is noteworthy that for the latter parameter, we considered all blue spots distributed on the yolk surface to avoid any bias between groups. A concentration of blue spots that likely corresponds to the blastoderm is clearly visible, while blue spots are also sporadically distributed on the yolk surface.

**FIGURE 4:**
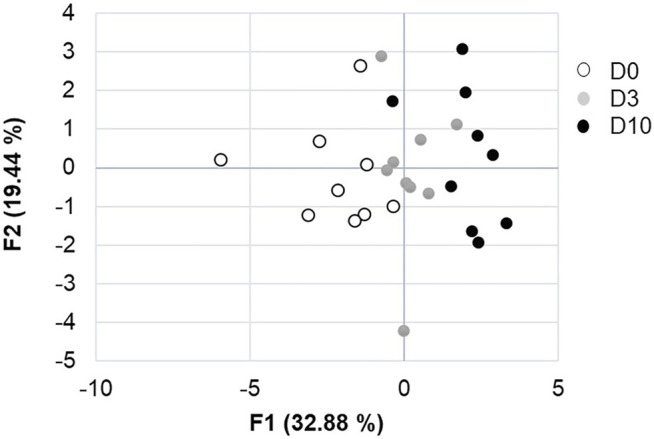
Analysis of principal components of the effect of storage duration on egg parameters. F1 and F2 axes explain 52.2% of the variability between D0, D3 and D10 groups.

CT images that are representative of each egg storage group (D0, D3 and D10) are shown in Figure 3. Analysis of principal components clearly demonstrated that all three groups of eggs, freshly laid (D0) or stored for three or 10 days (D3 and D10, respectively) were distributed distinctly (Figure 4). The D3 group was intermediate between the D0 and D10 groups, as expected. Combined results from CT and egg quality parameter measurements are presented in Table 2. No statistical difference was observed for egg weight, eggshell strength, yolk colour, yolk weight and egg white volume. Yolk index, yolk volume and Haugh units tended to decrease over storage, while egg white pH and the volume of the air chamber increased. Although not statistically significant between days of storage (*p* = 0.129, ANOVA test), the volume of the “grey zone” (that includes the blastoderm) tended to increase over time. The trend was confirmed when comparisons were performed between two groups (Mann-Whitney test). The difference between D3 and D10 groups was shown to be significant (*p*-value = 0.031), not significant between D0 and D3 groups (*p* = 0.297), and close to statistical significance between D0 and D10 groups (*p* = 0.060). It is noteworthy that volumes of the yolk and the “grey zone” were expected to be negatively correlated: the more pixels attributed to the “grey zone”, the less pixels assigned to the yolk.

**TABLE 2:** Effect of egg storage on egg parameters.

	Storage duration (days)			*p* Value	Trend during storage
	D0	D3	D10		
Egg weight (g)	56.73 ± 2.29	56.35 ± 2.05	56.05 ± 2.21	0.787	—
Eggshell strength (N)	37.35 ± 5.82	39.51 ± 6.19	34.85 ± 4.06	0.196	—
Yolk index	0.43 ± 0.03^a^	0.41 ± 0.03^ab^	0.30 ± 0.03^b^	**0.023**	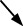
Yolk colour	6.27 ± 0.48	6.25 ± 0.37	6.35 ± 0.44	0.935	—
Egg yolk weight (g)	15.53 ± 0.99	15.82 ± 0.78	16.26 ± 0.74	0.130	—
Haugh units	87.30 ± 4.34^a^	80.01 ± 3.06^b^	74.57 ± 0.44^c^	**<0.0001**	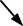
Egg white pH	8.53 ± 0.32^a^	9.20 ± 0.08^b^	9.32 ± 0.09^c^	**<0.0001**	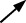
Yolk (cm^3^)	10.68 ± 0.94^a^	10.31 ± 0.78^ab^	9.61 ± 0.99^b^	**0.048**	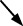
Egg white (cm^3^)	34.24 ± 1.53	34.27 ± 1.63	35.11 ± 2.08	0.636	—
Air chamber (cm^3^)	0.27 ± 0.07^a^	0.52 ± 0.07^b^	0.79 ± 0.17^c^	**<0.0001**	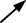
Blastoderm (cm^3^)	0.27 ± 0.11	0.28 ± 0.05	0.33 ± 0.06	0.129	—

Values with different letters indicate statistical differences between eggs stored for zero, three or 10 days (D0, D3 and D10, respectively; *p* < 0.05). As data normality was not observed for yolk index, egg white volume and eggshell strength (Shapiro–Wilk test), statistical analyses were performed using the Kruskal–Wallis test. Significant p Values are indicated in bold.

### Impact of Egg Storage for Three or Ten Days on Embryo Viability and Development

The time of storage prior to incubation did not significantly affect fertility (98.1% for the D3 group and 98.5% for the D10 group). However, when considering the whole period of experimentation (from EID0 to EID15), the duration of storage was shown to impair embryo viability: 4.7% mortality on the 65 viable D10-eggs vs. 1% mortality on the 105 viable D3-eggs.

**FIGURE 5:**
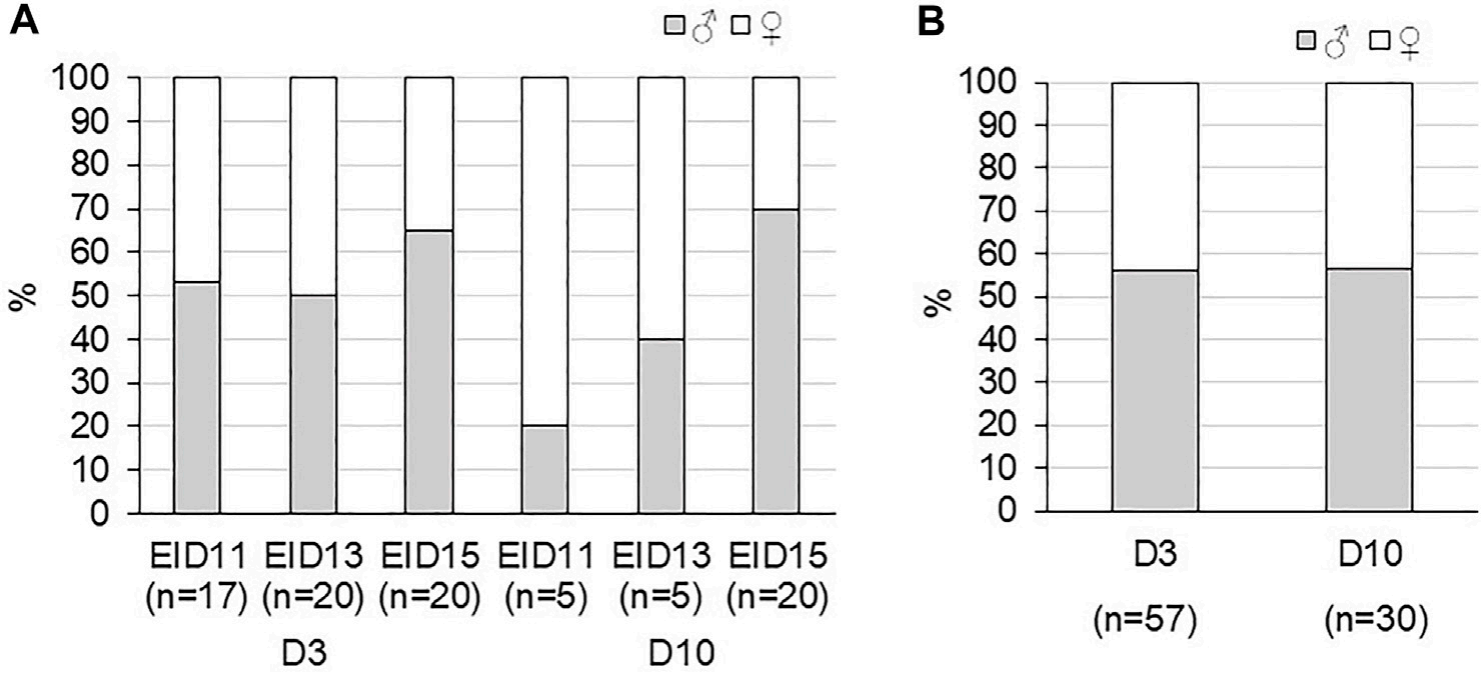
Sex ratio of fertilised eggs during incubation after three or 10 days of storage (D3 and D10, respectively) after 11 (EID11), 13 (EID13) and 15 (EID15) days of incubation. **(A)**. Sex ratio at each stage of incubation. **(B)**. Sex ratio in D3 and D10 groups (considering all EID11, EID13 and EID15 eggs).

In addition, the sex ratio determined on viable eggs was not equilibrated, especially at EID11 and EID15 after 10 days of incubation (Figure 5A). However, due to the small number of eggs analysed, we could not conclude on the effect of storage on sex ratio at each incubation day. After 15 days of incubation, the sex ratio of the 20 viable eggs was in favour of males (65 and 70% for D3-EID15 and D10-EID15 groups, respectively). Analysis of D3 and D10 groups (after combination of EID11, EID13 and EID15 eggs) indicated that sex ratios were comparable between the two groups, with a slight predominance of female embryos, regardless of the storage time (56/57% females vs. 43/44% males, Figure 5B). Therefore, from this experiment and considering the entire incubation period studied (EID11 to EID15), we concluded that there was no effect of storage duration on the sex ratio of the embryos.

**FIGURE 6:**
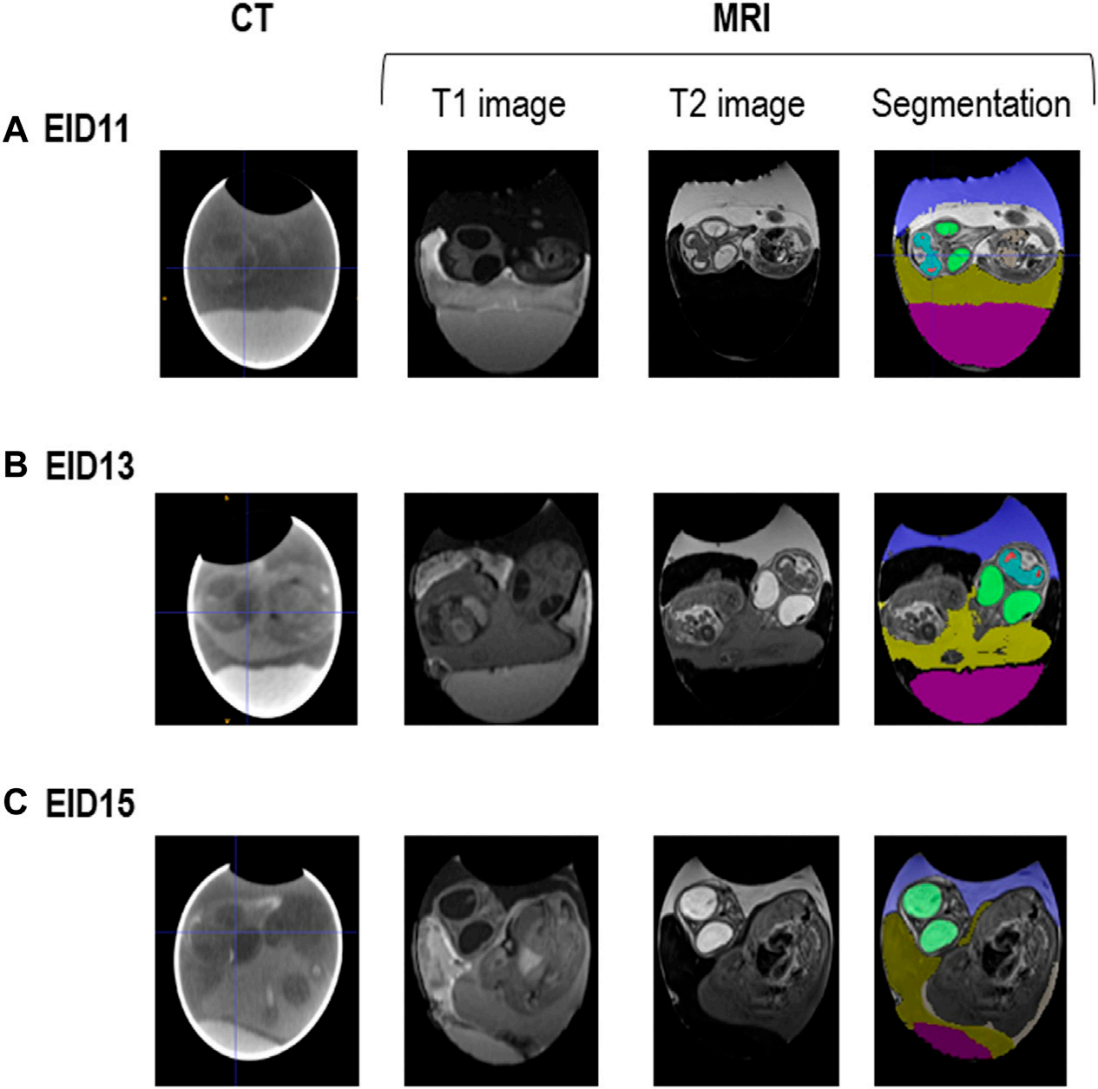
CT and MRI representative images of fertilised eggs during incubation. **(A)**. After 11 days of incubation (EID11). **(B)**. After 13 days of incubation (EID13). **(C)**. After 15 days of incubation (EID15). For segmentation (right panel), colours are as follows: in green, eyes; in blue and red spots, brain; in dark blue/purple, allantoic fluid; in pink, egg white; in yellowish colour, yolk. The amniotic fluid corresponds to the white zone between the embryo and the allantoic fluid on A (EID11, right panel) and to the dark zone between the embryo and the allantoic fluid on B (EID13, right panel). The change in the MRI contrast of the amniotic fluid between EID11 and EID13/EID15 (white to black), is due to the transfer of egg white into the amniotic cavity from EID12 onwards.

MRI images were analysed to measure volumes of internal egg components and the embryo (Figure 6).

Independent of storage time, the volumes of egg white, air chamber, allantoic fluid were shown to decrease over time (*p* < 0.05) during embryonic development, while those of eyes, brain and embryo increased (*p* < 0.05) (Table 3).

**TABLE 3:** Effect of egg storage on embryonic development.

Method	Volume (cm^3^)	Storage Duration (days)						Trend between D3 and D10 groups
		D3			D10			
		Incubation (days) EID11 EID13 EID15			Incubation (days) EID11 EID13 EID15			
CT	Egg white	9.22 ± 0.93	6.12 ± 1.07	2.93 ± 0.96	ND	ND	3.19 ± 0.86	—
	Air chamber	3.02 ± 0.29	3.70 ± 0.53	4.24 ± 0.53	ND	ND	4.23 ± 0.44	—
MRI	Allantoic fluid	10.97 ± 0.84	7.85 ± 0.69	5.08 ± 0.95	11.04 ± 0.51	8.35 ± 0.47	5.86 ± 1.67	—
	Amniotic fluid	3.42 ± 0.25	ND	ND	3.41 ± 0.26	ND	ND	—
	Eyes	0.38 ± 0.03	0.53 ± 0.04	0.59 ± 0.05^a^	0.35 ± 0.01	0.48 ± 0.03	0.54 ± 0.03^b^	D3>D10
	Brain	0.34 ± 0.01^a^	0.50 ± 0.03	0.73 ± 0.03^a^	0.28 ± 0.02^b^	0.47 ± 0.03	0.70 ± 0.02^b^	D3>D10
	Embryo	6.00 ± 0.40^a^	ND	ND	4.83 ± 0.30^b^	ND	ND	D3>D10
	Embryo + Yolk	ND	16.84 ± 1.13	25.08 ± 1.61^a^	ND	14.98 ± 0.99	22.12 ± 1.59^b^	D3>D10

Values with different letters indicate statistical differences between eggs stored for three (D3) vs. 10 days (D10) (*p* < 0.05). ND: not determined due to reduced number of eggs (CT, EID13 and EID15, D10) or technical constraints (amniotic fluid at EID13 and EID15; embryo at EID13 and EID15). n = 10, except for MRI, analyses of D10-EID11 and D10-EID13 eggs (n = 5 for each group). A Kruskal–Wallis test was first performed followed by a Mann–Whitney test for comparison between D3 and D10 groups. Values with different letters indicate statistical differences between eggs stored for three (D3) or 10 days (D10) (*p* < 0.05). Where statistics revealed significant differences between D3 and D10 groups, the trend is indicated in the last column of the table.

Pair comparisons between D3 and D10 groups at each developmental stage revealed a decrease in eye, brain and embryo (or embryo + yolk for EID13 and EID15 stages) volumes at EID11 and EID15 for D10 eggs, compared with D3 eggs (Table 3). Similarly, these embryo volumes at EID13, tended to be lower in the D10 group compared with the D3 group, although no statistical difference was observed (Table 3). Collectively, these data support that the embryonic development is delayed after a 10-days storage but not altered (absence of visible malformations).

**FIGURE 7:**
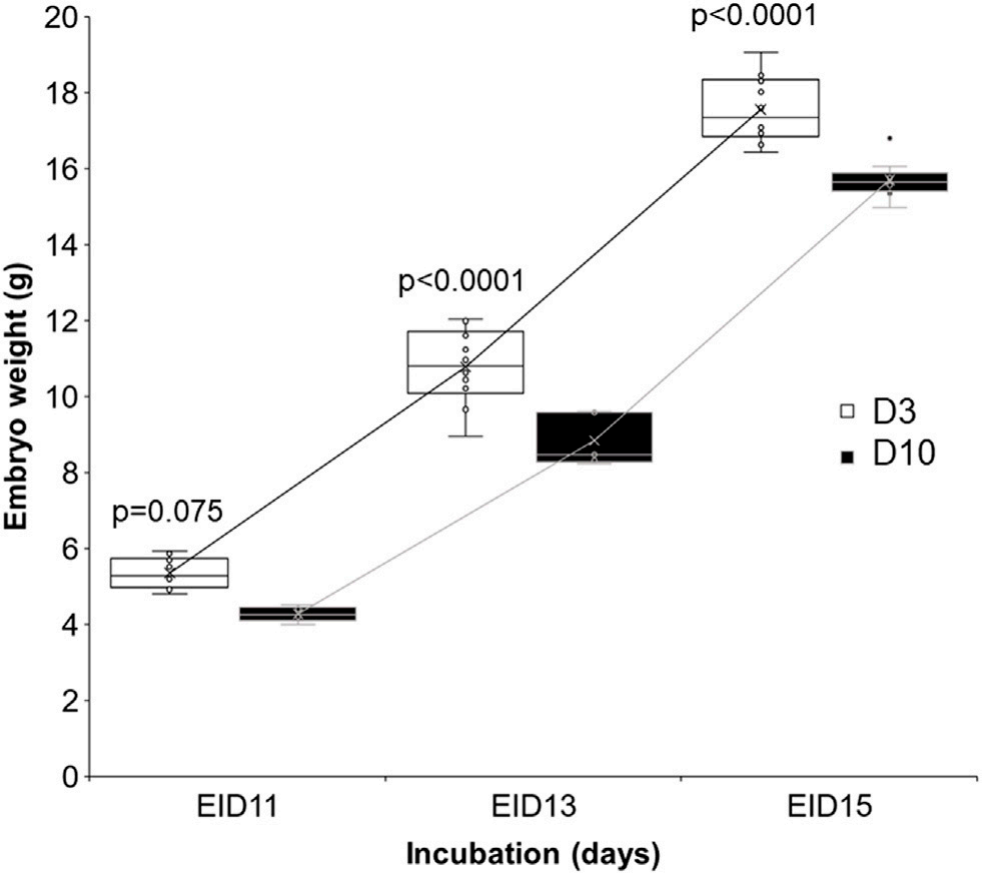
Effect of storage time on embryo weight after 11, 13 and 15 days of incubation. D3 and D10 eggs were opened at EID11, EID13 and EID15 after CT or MRI analyses and weighed. Statistical analysis was performed using one-way ANOVA with *p* < 0.05.

Embryo weight increased similarly between D3 and D10 groups (Figure 7). However, embryos from D10 eggs were significantly lighter than embryos from D3 eggs, especially after 13 (EID13, *p* < 0.0001) and 15 (EID15, *p* < 0.0001) days of incubation.

## Discussion

In birds, the embryo can pause its development until incubation, when the temperature is too cold or until the clutch size is optimal for brooding. This dormancy is characterised by cell arrest in the G (2) phase and suppression of apoptosis (Ko et al., 2017). However, extended storage impairs hatchability due to its deleterious effects on blastoderm viability. The detrimental effect of extended storage (over 7 days) on egg quality, embryo mortality and hatchability has been extensively reviewed in the literature (Asmundson, 1947; Brake et al., 1997; Fasenko, 2007; Bakst et al., 2012; Bakst et al., 2016a; Branum et al., 2016; Abioja et al., 2021; Melo et al., 2021; Özlü et al., 2021). Such negative effects are very well known to turkey and chicken breeders but they can have difficulties avoiding long storage practices for logistical reasons. Prolonged egg storage alters many egg quality parameters and may deteriorate the blastoderm to the point that it cannot resume upon incubation. Several studies have reported a decrease in viable blastodermal cells after storage over 8 days (Bakst et al., 2012). To mitigate such a negative effect of prolonged storage, strategies have been developed. These include short periods of incubation during egg storage (SPIDES) that reactivate embryo metabolism prior to incubation, thereby increasing the viability of embryos until hatching (Dymond et al., 2013; Damaziak et al., 2018). Some authors also reported a positive hatchability effect of storage at cooled temperatures [15°C, (Bakst and Gupta, 1997); 11.6°C (Guinebretière et al., 2022)], egg turning (Melo et al., 2021) or of *in ovo* injection of biological buffers to reinforce the buffering capacity of the albumen (Akhlaghi et al., 2013). The mechanisms underlying the reduced viability of blastodermal cells during storage have been partly elucidated: increases in expression of genes associated with apoptosis, oxidative stress and fatty acid metabolism (Hamidu et al., 2011; Bakst et al., 2016b). However, there are only few studies that investigated the impact of prolonged storage on the development of viable embryos. Some authors have described a reduced femur and tibia length being positively correlated with the duration of preincubation storage (Yalcin and Siegel, 2003), while others reported an altered growth of heart and liver (Christensen et al., 2002) and a slower metabolic rate (Christensen et al., 2001; Fasenko, 2007). In this study, we used non-invasive imaging approaches (MRI and CT) to investigate the impact of prolonged storage on egg quality, and on the growth and development of the embryo. Such imaging methods have previously been used to assess egg quality (Hutchison et al., 1992), to identify the localisation of the blastoderm (Bartels et al., 2008) and to monitor brain, liver or eye development (Bain et al., 2007; Boss et al., 2008; Zhou et al., 2015; Lindner et al., 2017).

We first explored how storage duration at 16°C, 80% relative humidity (classical hatchery conditions) for zero, three and 10 days affects egg quality. We used a combination of non-invasive CT on intact eggs and classical measurements on internal egg quality traits after egg breakage. In accordance with numerous articles and reviews (Benton and Brake, 1996; Brake et al., 1997; Abioja et al., 2021), we showed that the length of storage affects several egg parameter traits including egg white pH, Haugh units, yolk index and air chamber volume (Table 2). The volume assigned to the blastoderm tends to increase upon storage, especially when comparing three and 10 days of storage (Mann–Whitney test, *p* = 0.060), which corroborates previous studies (Bakst et al., 2012; Abioja et al., 2021). The analyses of the 30 eggs (10 eggs at D0, D3 and D10) revealed that the position of the blastoderm is always localised at the surface of the yolk, roughly in the middle (Figure 3), when the large end of the egg is maintained on the top (air chamber located at the top). Only one egg exhibiting a blastoderm oriented towards the bottom was noticed. It would have been interesting to put this egg back into the incubator to assess how this specific orientation impacts embryo development and whether it was still associated with a viable chick or not. Anyway, this observation suggests that CT imaging of eggs before storage to verify the localisation of the blastoderm may help the development of new experiments to determine whether this parameter (blastoderm location) is a predictor of hatchability.

Knowing that the increase in blastoderm volume is unlikely correlated with cell proliferation or the number of viable cells (Bakst et al., 2012), we hypothesised that it may correspond to the dispersion of the blastoderm cells as the vitelline membrane is losing its mechanical strength. Such a hypothesis is in accordance with the decrease of yolk index (Table 2) and the changes in yolk/white physicochemical properties. Further studies on the impact of extended storage (egg white physicochemistry, ultrastructure of the vitelline membrane that supports the embryo) may be useful to identify the determinant parameters that alter embryo survival and restarting. We believe that the quality of the vitelline membrane is crucial in the early stages of incubation as its inner layer contains many proteins that are assumed to support the development of the embryo and the expansion of the yolk sac during embryogenesis (Brégeon et al., 2022). Alteration of the perivitelline membrane is supposed to be a key determinant that can explain very early mortality. Although the duration of storage is detrimental to embryo survival, it is noteworthy that incubation of freshly laid eggs (that are characterised by high viscosity and neutral pH) is not correlated with higher embryo survival (Benton and Brake, 1996). Altogether these data highlight the major role of egg components that surround the blastoderm, on cell survival and hatching success. This observation is partly corroborated by our data, where storage up to 10 days at 17°C, 80% RH did not affect fertility (the number of eggs that restarted was comparable between D3 and D10 eggs) but impaired embryo viability (4.7% mortality for D10 eggs vs. 1% mortality for D3 eggs during incubation). The reason why some eggs resist extended storage more is not known but it probably results from many variables including genetics, egg quality (including composition) and embryo specificities. Christensen et al. reported that embryos from a genetic line that resisted storage mortality maintained greater glycogen concentrations in muscle and heart tissues than those from a line and old hens associated with reduced survival rates (Christensen et al., 2001).

Hence, similar to MRI (Hutchison et al., 1992; Klein et al., 2002; Burkhardt et al., 2011), CT imaging can help to localise the position of the blastoderm, as confirmed in this study and others (Bartels et al., 2008), which might be particularly interesting to develop tools that require information from the embryo. For example, identifying the exact position of the blastoderm on the yolk may help in developing sexing methods, as suggested previously (Burkhardt et al., 2011) and to differentiate an early-dead embryo from an unfertilised germinal disc (Bakst et al., 2016a). In this study, CT has been used to estimate the localisation of the blastoderm during storage but it might be interesting to further explore how the CT signal associated with the blastoderm is changing during the first 3 days of incubation, and whether this method can be used to monitor early stages of embryonic development.

Interestingly, the sex ratio of viable embryos was shown to be comparable between the two experimental (D3 and D10) groups (Figure 5). The verification of this parameter was essential to avoid any bias associated with the sex of the embryo, knowing that the maturation/growth of the embryo may differ between males and females, even during early stages (Tagirov and Golovan, 2015; Hirst et al., 2018). Such information remains important in the context of the development of methods to avoid the culling of male chicks. Indeed, storage or incubation methods that could imbalance the sex ratio in favour of females would diminish the number of male embryos and chicks to eliminate (Gautron et al., 2021).

Data related to embryo weight and brain, embryo and eye volume (Table 3; Figure 7) all support that storing eggs for 10 days negatively affects embryo growth. Similar to our results, body mass of EID15 embryos was previously shown to be significantly affected by a storage duration of up to 3 weeks (*p* < 0.001) (Branum et al., 2016). It was also reported that the acid-base balance of embryos was modified according to storage duration (Branum et al., 2016). However, storing eggs for 10 days does not seem to affect the kinetics of embryo development (similar growth curves, Figure 7). Further studies on a higher number of eggs are needed to increase the statistical significance of some parameters including egg white and allantoic fluid volumes (Table 3). Notably, at EID15, the egg white volumes tended to be higher in D10 eggs than D3 eggs (2.93 ± 0.96 and 3.19 ± 0.86 in D3 and D10 eggs, respectively). A similar trend was observed for the volume of allantoic fluid, which was higher in D10 than D3 eggs (5.08 ± 0.95 and 5.86 ± 1.67 in D3 and D10 eggs, respectively). These observations corroborate the aforementioned conclusion that the growth/developmental stage of D10 eggs was delayed compared with D3 eggs. Indeed, the decrease in egg white volume, which is located at the bottom of the egg, is concomitant with its transfer into the amniotic cavity between EID11 and EID12 (Da Silva et al., 2019). From EID13 onwards, the amniotic fluid/egg white mix will start to be absorbed orally by the embryo as a source of amino-acids, to accompany its growth. The higher volume of egg white noticed in D10 eggs compared to D3 eggs suggests that the egg white transfer into the amniotic cavity was also delayed in D10 eggs.

Further research should include analyses of other physiological/phenotypical traits including organ growth, embryo positioning within the egg and initiation of skeletal mineralisation, to complete the story and facilitate the identification of indicators of normal or abnormal development/growth. To our knowledge, only a few articles report such experimental studies (Christensen et al., 2001; Christensen et al., 2002; Yalcin and Siegel, 2003; Fasenko, 2007). Our data suggest that the physiological stages of EID11, EID13 and EID15 embryos are more advanced for D3 eggs than D10 eggs and that the development of D10 embryos is delayed compared with D3 embryos. Although our experimental design did not include incubation up to hatching, other studies reported that chicks originating from eggs stored for a long period hatched later than embryos from eggs stored for a short period (Christensen et al., 2002; Tona et al., 2003a; Tona et al., 2003b). However, we have no evidence to date that the duration of storage induces abnormalities.

Additional data on the volume of embryo organs and supporting structures, the orientation of the embryo inside the egg and the movements of egg structures during incubation should also help to revise the atlas of chicken development (Hamburger and Hamilton, 1951). Indeed, most modern chicken lines have been selected for decades on performance, egg or meat quality, and there is an increasing number of articles that alerts on differences in metabolism and health between commercial genotypes (Koenen et al., 2002; Tona et al., 2004; Buzała et al., 2015). Comparisons of the embryonic development (organogenesis, growth, kinetics) between several contrasted phenotypic lines should help to investigate the impact of genetic selection on embryos, whose proper development predetermines health and welfare of chicks and adult chickens. Integrative studies considering the normal development of the embryo using several levels of structural organisation (from the embryo within the egg to the molecular profiling of egg/embryo contents) are lacking. New data from experiments addressing the impact of genetics, age and nutrition of reproductive hens, egg storage and incubation conditions on egg and embryo specificities may be useful for the development of predictive tools (Yimenu et al., 2017) to model egg quality (for both table and fertilised eggs) and developmental kinetics of embryos, upon exposure of eggs to suboptimal conditions.

## Conclusion

In this work, we demonstrated: 1) that the storage of fertilised eggs up to 10 days is associated with a decrease in several egg quality parameters; and 2) that the development of embryos exposed to extended egg storage is delayed compared to those stored for a shorter period. These data combined with published works suggest that eggs exposed to different storage durations are likely associated with differences in embryo maturity and hatching time. This observation underlines the necessity to improve the homogeneity of egg batches in terms of storage conditions to narrow the hatching window, thereby limiting the time spent by new hatched chicks in hatchers (without access to water and food). This work also evidences the relevance of imaging techniques to monitor the development of bird embryos during incubation but also to visualise and quantify how egg components (egg white, extraembryonic fluids) are modified throughout incubation. Classical measurements of egg quality parameters and embryo development usually require the egg opening and embryo killing. In this respect, CT and MRI techniques are non-invasive approaches. Although some egg quality parameters cannot be determined using these techniques (egg white pH and viscosity, yolk colour and index), they facilitate the analysis of some egg and embryonic components that are usually difficult to measure/evaluate (volume of the air chamber and extraembryonic fluids, embryo positioning, and movement of egg structures during storage and incubation).

## Data Availability

The raw data supporting the conclusions of this article will be made available by the authors, without undue reservation.

## Abbreviations

CT, computed tomography; D, day; EID, embryonic incubation day; MRI, magnetic resonance imaging; RH, relative humidity.
